# Position Paper Progress in the development of biomimetic engineered human tissues

**DOI:** 10.1177/20417314221145663

**Published:** 2023-02-27

**Authors:** Umber Cheema

**Affiliations:** Division of Surgery and interventional science, UCL Centre for 3D models of Health and Disease, Fitzrovia

**Keywords:** Tissue engineering, tissue models, bioengineering, regenerative medicine

## Abstract

Tissue engineering (TE) is the multi-disciplinary approach to building 3D human tissue equivalents in the laboratory. The advancement of medical sciences and allied scientific disciplines have aspired to engineer human tissues for three decades. To date there is limited use of TE tissues/organs as replacement body parts in humans. This position paper outlines advances in engineering of specific tissues and organs with tissue-specific challenges. This paper outlines the technologies most successful for engineering tissues and key areas of advancement.

## What is TE for?

Engineered human tissues to replace body parts is the most common end-point associated with TE. TE has also resulted in the development of biomimetic tissue models which have given us insight into disease progression and mechanistic understanding of tissue development. A further use of engineered tissues is to measure response to novel drugs and therapies for specific diseases. The requirements of engineered tissues are different depending on the expected use.

For an engineered tissue as a replacement body part the function of the overall tissue is key. For TE skin, this would be the primary function as a barrier to external factors with mechanical features to allow extension in multiple directions. Skin also facilitates and hosts sensory nervous input. Furthermore, skin also hosts specialised cells like melanocytes for melanocyte regulation which is a key function of skin. In direct comparison to engineering a complex skin model in its entirety, any TE skin model for use in research would need to clearly define the specific and relevant outcome parameters needed to be observed or measured to validate the model. An example for TE skin may be the spread of melanoma. This would require a model to test the growth of melanoma in skin, and the interaction of this with healthy stromal cell populations found within skin specifically to interpret response of the melanoma to a novel drug/treatment.

3D tissue models should always be as simple as possible without losing key parameters deemed critical to study specific functions. Therefore, the parameters dictating which biomimetic features need to be recapitulated in engineered tissue are different dependent upon whether they are body part replacements or tissue models to test drug response or other therapies. It is important to define the limitations of tissue models. Each model is used for specific purposes and therefore not all tissue complexity needs to be recapitulated. It is therefore pertinent to define requirements based on the eventual use of the engineered tissue (Table 1).

**Table 1.** A table outlining some of the key requirements for engineered tissues as replacement tissues in the body or as biomimetic tissue models for *in vitro* use.

Key requirements – what is needed	
Replacement tissue – implantation	Tissue models – *in vitro*
Tissue/organ function Matching matrix/composition Matching material/mechanical properties to tissue *in situ* Remodelling of tissue by host Integration to key systems (vasculature, nervous, immune)	Cell survival in biomimetic 3D template Recapitulation of biophysical features found in tissue *in situ* Measurable outcomes parameters to test disease progression and drug targets Reactivity to stimuli (physical and chemical, including toxicity) Reproducibility (medium-high throughput) Low cost Definition of limitations

## Key considerations of the tissue

**Figure 1.**
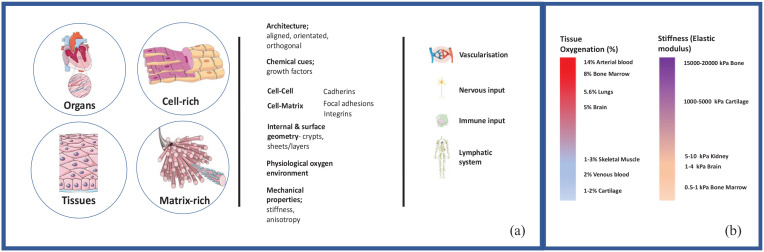
(a) Four key considerations for tissue engineered (TE) products and models. For effective tissue engineering considerations of whether the tissue is a tissue or organ, whether it is cell-rich or matrix-rich, the constituent biophysical parameters of the native tissue and the necessary support systems required to integrate tissues into the body. Each element/requirement must be carefully considered and crafted into any products to truly recapitulate the native tissue. (b) The tissue oxygenation and tissue stiffness (elastic modulus) for different tissues.^9–11^ Figure was generated using Servier medical art.

Tissue engineering is a multi-disciplinary field spanning the biological, chemical and physical sciences. The aim is to build functional and structurally biomimetic tissues either to replace human tissues, act as immature tissues to aid the regeneration of diseased/unhealthy tissues *in vivo* or engineer functional tissue models to be used to testing therapeutic intervention and targets. To engineer biomimetic functionality and biomimetic structural organisation, multiple aspects of tissue design need to be controlled (Figure 1).

The problem is that regardless of how simple we consider a tissue to be, it is integrated with complex systems and is reliant upon other tissues. Many tissues have transitional elements, for example tendon connects skeletal muscle to bone, at one end the tendon tissue gradually becomes mineralised (the bone end) and at the other end it contains interdigitating tendon fibres and myofibres (the muscle end). Engineering this complexity to account for the integration of tendon into two different tissue types is challenging. A further example is ligament. This is a collagenous tissue, relatively avascular containing ligament cells which have a slow turnover. Yet, this tissue links bones together for a coherent skeleton, and thus has a transitional tissue component where the ligament gradually becomes bone. This needs careful engineering, particularly as this tissue is under constant mechanical loading which is key to maintenance of cellularity and tissue architecture. Generating ligament tissue *in vitro* has been hard because of the strength of the tissue, which is under dynamic mechanical loading, reliant upon its orientated and dense collagen architecture. Currently, the gold standard for ligament repair is autograft.

Tissues comprise of a cellular component and a matrix component. Cells are the living biological units which control both their function and their immediate biophysical microenvironment, including the matrix. Thus providing cells with appropriate cues to direct their biological function is a key consideration we need to make. An emerging concept is the premise that a scaffold or matrix is not simply a 3D structure into which cells grow, but that the scaffold or matrix are tissue specific templates, which may or may not perform a mechanical role, but what is key is that they direct and guide cell behaviour in a biomimetic manner.^1^ 3D environments provide cells with a rich environment which promote cell–cell and cell–matrix interactions.^2^ Furthermore, the attachment of cells to native extracellular matrix (ECM) components provide sites for cell attachment and the subsequent expression of integrins and receptors in response to binding, methods for signal conduction and physical stimulation to initiate feedback loops to promote cell-generated ECM production.

Cell-rich tissues contain specialised cells which regulate the adequate functioning of the organ, but these cells are also involved in controlling their physical microenvironment through remodelling and regulating other systems including vasculature and immune-competency. These cell-rich organs are highly vascularised due to the high cellular demand for oxygen and nutrients. These organs generally have a low composition of matrix which is nevertheless key to function and important as it provides physical cues which can guide specific cell behaviour. An example is a study which compared neural network activity from primary neurons co-cultured with glia grown on different types of ECM coatings and it was found that the presence of ECM accelerated the formation of networks between these cell populations.^3^

In matrix-rich tissues, including bone and skin, the high composition of matrix is not only critical for the key mechanical roles these tissues play, but the matrix also acts as a template to direct cell function. Through the very nature of tissues being 3D, this concept of the template is a crucial foundation for any TE approach; however, tissue specificity is crucial to consider as the template needs to replicate biophysical features which simulate the native microenvironmental stimuli of the tissue in question.

The use of native proteins as templates to direct and grow engineered tissues poses additional complexities due to the difficulty in controlling multiple facets of native protein architecture. Collagen I is the predominant matrix protein of most tissues of the body and yet controlling the density, the fibril diameter and orientation is extremely difficult. Methods to control collagen alignment include the use of magnets and fluid flow.^4,5^ However, these methods also direct and influence cellular behaviour and also that of any supplementary proteins deposited by cells. Each and every engineering approach therefore directs multiple facets of an engineered tissue and subsequently influences multiple cellular and non-cellular processes downstream.

When engineering tissues, the approach is different for specific tissues. As an example, bone tissue, where mechanical fidelity is key to that tissue’s function, requires a greater emphasis to be placed on the mechanical properties of engineered bone tissue. As a direct comparison, in liver, one of the main roles of this tissue is to detoxify and aid digestion; therefore, the adequately functional hepatocytes is important for the tissue to fulfil its main role. Maintenance of high vascularity is therefore a key priority for retention of cell viability in cell-rich tissues.

For TE of tissues as replacement body parts, the integration of a tissue with other tissues is vital. The development of engineered vasculature, lymphatic systems and neural networks into tissues is one approach for successful integration with the host following implantation, but the other approach is to prime cells to recruit these systems from the host following implantation. Examples of this include stimulating host driven angiogenesis. Approaches taken to enhance this process include the addition of HIF-I regulating particles^6^ into engineered tissues, but a more physiological approach is to pre-condition cells in engineered tissues with low levels of physiological hypoxia to stimulate their natural production of angiogenic growth factors, including HIF-I.^7^

Integration of TE constructs is a key consideration both for TE body part replacement as well as integration of tissues into complex model systems. Integration is hierarchical in that many layers of integration need to be considered and these are specific to TE for replacement of body parts or as tissue models.^8^ For replacement body parts, the primary goal is physical integration of engineered tissues into the body which is mainly done by adhesion, bonding or even suturing. However, over time further layers of integration are required if the TE construct is to be functional in any sense of the word. Cells and matrix need to work together with existing systems, for example an engineered piece of skin must integrate with surrounding host skin vasculature. Furthermore, this tissue needs to be mechanically sound and match the material and mechanical properties of the surrounding tissue. In the case of skin elasticity is a key material feature which is necessary for the appropriate biomimicry of the tissue.

## TE approaches

For all TE, a suitable scaffold/matrix/template needs to be identified. This template plays a role in reproducing key aspects of the biophysical micro-environment or niche that cells in the native tissue are exposed to. The geometry, stiffness (including haptotactic gradients), matrix composition (specific integrin expression for binding), mechanical stimulation, hypoxic environment are all aspects of the biophysical stimuli which control cell behaviour within a defined tissue niche. Controlling any single or multiple aspects of the biophysical microenvironment is difficult and co-dependency means often virtually impossible for every feature to be controlled in TE. Our reliance upon cells self-regulating and remodelling the environment in the native tissue is appreciated within the field. A clear example is the understanding that cells themselves, including mesenchymal stem cells, are trophic factories capable of producing cytokines and growth factors in response to certain biophysical and chemical stimuli.^12^

A traditional and accepted facet of TE is the role of the scaffold or template in directing and controlling cell behaviour and fate. The main templates used are the following; (i) self-assembly of cells in 3D and cell-generated matrix deposition (spheroids, hanging drop cultures); (ii) culturing cells/tissue explants in 3D scaffolds (native matrix, decellularized tissue matrix (human or xenogenic), natural matrix (alginate, chitosan, etc), synthetic scaffolds and composites); (iii) 3D printing and bio-printing of cells with different matrix compositions; (iv) organ on a chip culture, including multi-organ on a chip.

Each of these approaches offers unique advantages for engineering tissues. A clear example is the engineering of polymer scaffolds which allow for the controlled imposition of specific material properties. Polymer manufacture can be tailored to generate polymers with specific material properties; however, this is using synthesis materials which may have biocompatibility issues. Although a significant body of research exists to show that synthesis scaffolds can be engineered to be biocompatible, cells will attach to native proteins and extracellular matrix (ECM) in unique ways through the expression of specific subsets of integrins and focal adhesions, this is tricky to replicate in an exact manner with synthetic scaffolds.

The use of native scaffolds or templates in which to culture cells recapitulates a more native environment to stimulate cell attachment and cell function. However, native proteins and ECM are difficult to handle due to limitations in our ability to control native protein structure including aspects such as size, crosslinking, topology, elasticity, and orientation.^13^ Control of these parameters is eloquently and exquisitely mastered in the body in both development and repair of tissues/organs *in situ*. As researchers, we have developed methods to control single aspects of ECM architecture, but simultaneously controlling them all is not yet possible and may never be.

Another key aspect to consider is how we assess tissue models for testing drugs and other therapeutic interventions for tissue-specific diseases and conditions. Without clear and measurable outcome parameters of assessing disease progression, a tissue model will not fulfil its potential for use as a test bed for novel and emerging therapeutic approaches. A clear example is the TE of cancer models for the prediction of response to chemotherapeutic drugs and emerging therapies including immune-therapies and novel drugs. The most common approaches to assessing efficacy of an intervention is cell death, however what is more pertinent is cancer-specific cell death compared to normal healthy stromal tissue death. Many 3D TE cancer models lack the complexity of both the cancer and normal stroma. There are notable exceptions where both tumour and stromal tissues have been engineered.^14,15^

## TE as tissue replacement or as a 3D tissue model

### Skin tissue replacements

Tissue engineering research has come a significant way with insights into complex developmental processes and organ development deciphered through using TE technologies. TE of skin is an example where significant advances have been made. Skin is a complex, multi-layered and multi-cellular tissue. It comprises epidermis, dermis and subcutaneous tissue layers, each with specific cell populations and specific tissue architecture. It is well vascularised and plays an important role as a sensory organ.

There are multiple TE products, mainly for diabetic ulcers and other large skin wounds, which are currently available.^16^ Many FDA-approved skin substitutes in the market contain allogeneic cells, where a common source of fibroblasts and keratinocytes is form neonatal foreskin (Dermagraft ® Apligraf ®). Apligraf® uses bovine type I collagen matrices which are seeded with human neonatal fibroblasts in a dermal layer and keratinocyte cells in an epidermal layer. Once implanted, reports of wound healing have been good.^17^ Dermagraft® is a product where neonatal fibroblasts are seeded onto synthetic polymer meshes, ready for implantation. The layered structure of skin is not replicated and neither is the diverse population of cells. These products have shown some clinical significance in terms of wound healing.^18^ Theraskin® is cryopreserved human skin allograft which is biologically active. As it is an allograft, the natural layering of the tissue, epidermis and dermal layers are present, and different cell populations are maintained within the layers. This product is not necessarily engineered, but more akin to allogeneic tissue transfer with an additional cryopreservation step. There is an emerging appreciation by researchers in this field that the multi-layered complexity is challenging to engineer, especially with incorporation of skin specific features. Engineering multi-layered structures, with appropriate and biomimetic adhesion, remains a key challenge for TE skin. New generation products containing different cell populations found in skin, including mesenchymal stem cells are being developed, but have yet to be granted approval.^19^

### Skin tissue models

There are many successful TE skin models available. A clear breakthrough has been the ability to culture keratinocytes using an air–liquid interface with 3D scaffolds and the introduction of the microbiome into such skin models.^20^ There are organisations, including Labskin®, where support to use the Labskin skin model, is offered. Such models are mainly used for toxicology testing and testing of formulated products for different sectors including cosmetics and pharmaceuticals. The skin models used by Labskin recapitulate the layers of skin, so include an epidermis and dermis, with appropriate cells seeded in fibrin. More simpler engineering approaches, including the use of polycarbonate sheets to culture cells and form reconstructed human epidermis (Straticell®) are also used for toxicology testing. The trajectory of original technology development and research finding through to commercially available models in this sector has been roughly a decade.^21^ There are also emerging models which have gone on to introduce sensory nervous input and the addition of melanocytes which are necessary to model reactivity to specific stimuli.^22^ The key role of skin as a sensory organ means that without the incorporation of nervous input, the TE tissue does not adequately function as skin. This key challenge must be addressed and significant further research is required.

### Bone tissue replacements

TE of bone is a well-researched area. Due to the necessary demands of bone tissue, in terms of mechanical stiffness and strength required for adequate functioning, there is a justified emphasis on material properties of engineered tissue.^23^ Currently there are multiple TE bone grafts, including Actifuse® which is a Silicate substituted calcium phosphate, Vitoss® a bioactive glass and calcium phosphate and Healos® which is made up of cross-linked collagen fibres coated with hydroxyapatite into which autologous bone marrow can be added.^24^ The unique architecture of bone, which includes both trabecular and cortical bone can be recapitulated using 3D printing technologies, but the incorporation of multiple cell types with the biomimetic configuration found in bone is challenging to recapitulate. There remains a lack of convincing data on this.

Due to the mechanical properties needed for bone grafts and the ability of cells to repopulate implanted grafts, ethical issues with the introduction of cells form allogeneic sources have not been tackled. 3D printing or bio-fabrication methods offer opportunities to engineer TE bone implants with internal and external geometries as template features to direct further bone growth and integration with host systems including vasculature.

### Bone tissue models

There has been a significant drive to tissue engineer bone. As bone is a dynamic tissue and remodelled in response to changing demands on the tissue, TE models need to be appropriate to the appropriate phase and state of the tissue.^25^ Models for specific bone diseases, including osteoporosis, osteogenesis imperfecta and even osteosarcoma are extensive; however, it is important to come back to the key role the template or scaffold plays in directing cell behaviour and remodelling of the matrix.^26^ Given the major organic component of bone is collagen (deposited and remodelled by osteoblasts) which is the site for mineralisation by osteoblasts, recent models using dense collagen as a template for osteoblasts have shown the formation of woven bone (immature) within 21 days.^27^ *In vitro* models which have been used to study the actions of osteoblasts and osteoclasts have tended to use collagen as either the sole template component or at least as the main component.^28,29^ These models have successfully managed to recapitulate bone remodelling, as both the deposition and resorption of bone has been achieved in these models.

### Cartilage (articular) tissue replacement

Articular cartilage is predominantly composed of hyaline cartilage and significant work has been done in TE of this tissue. Articular cartilage is often found at the surface of bones between articulating joints with common areas being the knee, shoulder and elbow joint. Hyaline cartilage has unique properties which allow for low friction between the two surfaces of articulating cartilage allowing for smooth and uniform motion as well as resistance to compression and transmission of loads to underlying bone.^30^ The main ECM composition is collagen II and proteoglycans, which are able to control matrix hydration by trapping and holding water. The clinical need to replace cartilage is growing with an ageing population, but there is also a need following injury. Approaches to repair this tissue include micro-fracture at the site to initiate repair and also implantation of articular chondrocytes (ACI) either directly or within a matrix (MACI). However it has been shown that methods of cell delivery are only just as successful as micro-fracture.^31^ The most promising cell type for use in TE cartilage currently being developed are autologous mesenchymal stem cells in a variety of different matrices including the use of 3D printing.^32^ Allogeneic umbilical cord blood stem cells delivered in sodium hyaluronate have been approved for use in cartilage TE by CARTISTEM®, adding to the different cell types used for cartilage TE. The main issues identified with engineering of this tissue as a tissue replacement is the integration of the cartilage with the bone. If this integration is not successful, the biological and biomechanical properties necessary will be compromised.

### Cartilage (articular) tissue models

The main disease mechanisms studied utilising 3D tissue models of cartilage are osteoarthritis and rheumatic diseases. Osteoarthritis and other rheumatic disease affecting joints are not only a disease of the cartilage, but in fact a cumulative effect of all the tissues in the joint. Some of the most exciting models proposed incorporate all of the components of the joint including Joint-on -a-chip technology, where the multiple cell types are cultured as distinct ‘organs’ with the addition of media flow to transport signalling, physical/mechanical stimulation as well as other biophysical features.^33^ This highlights the need to consider all tissues within a specific disease and engineer systems where each tissue component and system is added.

### Skeletal muscle tissue models

There has been significant research in the area of skeletal muscle tissue engineering. It is possible to engineer aligned, multi-nucleated myotubes and myofibres, by culturing skeletal myoblasts in extracellular matrix components (including collagen and fibrin) validated through the measurable ability of these cultures to ‘twitch’.^34,35^ Furthermore work on the innervation of skeletal muscle models has shown promise in the development of mature skeletal muscle phenotype, as both contractility of muscle and cytoskeletal organisation are more biomimetic with the introduction of motor neurons.^36^

Although there is currently no tissue replacement for skeletal muscle, research into the prospect of engineered meat for human consumption is a clear deviation from the traditional purposes outlined in TE principles. There have been a multitude of TE approaches to engineer skeletal muscle as a meat equivalent for consumption, including the use of 3D bioprinting.^37^ Furthermore, biohybrid robots have demonstrated the addition of engineered skeletal muscle introduces the capacity to control movement through inherent contraction.^38^ TE of skeletal muscle clearly demonstrates how TE technologies can be used in diverse ways and may have impact in unique settings.

### Renal tissue models

Due to the worldwide problems of renal dysfunction and renal failure, the renal system is an important target for tissue engineers and bioengineers. Kidneys perform multiple roles including filtration of blood, secretion of key factors including endocrine and immunologic factors, and maintenance of homeostasis, which are all essential for the organ to be deemed functional. Given these multiple roles, kidney is a complex and multi-cellular organ with very specific structures in place. Engineering of this tissue poses unique challenges given the multiple cell types involved and the very specific microarchitecture and structures formed, including the proximal and distal tubules.^39^

There is no current bioengineered kidney or renal tissue component which is available for clinical use. However, the main approaches which have been considered for this tissue are 3D bioprinting, kidney organoid culture and the seeding of renal cells into decellularized matrices.^40^ In particular for renal tissue, 3D bioprinting has the distinct advantage of generating tissues in a layer-by-layer structure, where the precise deposition of matrix (biomaterial ink) and cells (bioink) can be controlled as the tissue in ‘built’ up. The exact structures of specific renal tissue can be fabricated and the limitations here are the actual bioprinting machines, which are being developed rapidly to form more and more complex, biomimetic structures.^41^

Progress in engineering kidney organoids has been rapid, including the recent development of the organoids with a recapitulation of the spatial assemblies found within the native kidney’s ureteric bud or collecting system.^42^ 3D branched morphologies recapitulating those found within adult kidneys can now be engineered in organoids.

### Cardio-vascular tissue models and heart valves

Tissue engineering cardiac tissue or even an entire heart is multi-faceted and different strategies have been used to TE specific components of this organ to support its function. The high cellularity, the physical demands on the tissue and organ, the constant dynamic flow and mechanics associated with this tissue and uniquely challenging.

### Cardiac patches:

*Tissue engineered cardiac tissue*: There is a growing need alongside a growing appreciation for the need of next-generation tissue-engineered heart valves to replace prostheses for surgical and transcatheter replacement, as well as replacement for allograft or autograft.^43^ Challenges to address with engineered heart valves include immune-compatibility, haemocompatibility, remodelling and growth capacity, and these have limited adoption of tissue engineered solution. The area of TE receiving most attention for cardiac tissue is decellularized tissues and due to the limitation of allogeneic tissue, much of this is from xenografts. As well as numerous preclinical studies using decellularized xenografts for heart valves, the most notable advancement was the porcine to human heart transplant in January 2022.^44^ Although the patient only survived for 2 months following transplantation, the removal of xeno-antigens by gene manipulation was critical as a proof of concept that an organ with functioning cells could survive within humans and perform organ specific functions. The aspect of TE in this is the genetic modification of cells to reduce the immune response by the host. This approach to reducing the human immune rejection response will play a key role in TE of other organs/tissue containing allogeneic or other cells.

### Gene therapy

The premise of gene therapy is that instead of delivering proteins and growth factors to treat disease and disease symptoms, resident tissue cells have genes delivered into them to alter cell translation of proteins. The successful uptake of genes or sequences by target cells allows for the translation of proteins or silencing of target genes associated with disease. This can be an advantageous strategy as a manipulation of existing cells, which will be non-immunogenic, is likely to result in more controlled production and release of proteins and growth factors, in spatial and temporal terms. There are disadvantages which are related to the delivery of vectors to non-specific cells. Delivering the correct gene to a specific region and allowing for a sufficient level of expression without causing adverse reactions poses challenges within the gene engineering field.^45^

The benefit of transporting genes and vectors to specific tissue sites through the use of 3D scaffolds holds a number of advantages. The first is that delivery scaffolds can be adequately designed to match and mimic the physical properties of tissues. This means that there is immediate tissue support, while factors are held in place. Furthermore the properties of delivery scaffolds can be designed to control aspects including the release rate of vector and the degradation of the scaffold for optimal delivery of genes. Gene editing within organoid cultures has also been used to generate specific disease models.^42^

TE models to test delivery and mechanism of action for immunotherapies are being developed. One facet of this is the engineering of biomaterials to effectively delivery immunotherapies. Similarly to gene therapy delivery, once a molecule is delivered in a 3D scaffold, which can be tailored to degrade at a specific rate, there is the added advantage of a much longer and sustained delivery of any therapeutic.^46^ Investigation of mechanism of action of specific immunotherapies, or the efficiency of a therapy can be done utilising TE in vitro models of the lymph node and its surrounding microenvironment. Lymph nodes are characterised by their distinct and complex organisation of cell types. The main approaches used to engineer lymph nodes to test immunotherapy is mainly 3D hydrogel-based culture systems as well as microfluidic chips.^47^

### Current TE market

There are currently 160 tissue engineering products and regenerative medicine products either with approval or in the late stages of development by companies located across the globe.^48,49^ Each product requires approval from the relevant jurisdiction, including most commonly the US Food and Drug Administration (FDA), European Medicines Agency (EMA) and the Pharmaceuticals and Medical Devices Agency (PMDA).

It can be somewhat difficult to derive numbers of tissue engineered products, the FDA for example, notes that the 2018 product list for Human Cells, Tissues, and Cellular and Tissue-Based Products has a total of 361, which will include TE products (https://www.fda.gov/vaccines-blood-biologics/tissue-tissue-products/fda-regulation-human-cells-tissues-and-cellular-and-tissue-based-products-hctps-product-list).

Furthermore, classification of products is slightly different dependent upon the area. TE products are a sub-section within the Advanced therapy medicinal products which are regulated by the EMA. Recent examples of TE products are Holoclar, classified as a tissue engineered product for the treatment of cornea damage through the implantation of autologous stem cells seeded on fibrin membranes.^50^ Additionally, Spherox is a tissue engineered product which delivers spherical aggregates of chondrocytes to damaged knee cartilage.^51^

### Where are we headed: Challenges and future directions

Tissue engineering products and biotechnology companies specialising in TE products are growing. Analysis of the public market in the US from 2011 to 2018 indicated 49 companies identified as tissue engineering companies, with 21 of those having tissue engineering products on the market.^52^

There is a need for more comparative studies between *in vitro* engineered tissue models and animal models. Drugs undergoing processing for approval for human use are currently tested using animal models to test for toxicity, biocompatibility and efficacy. If TE models are ever to be used instead of animal models, convincing comparisons must be made. TE models need to have clear, measurable parameters which can be used to test for (i) toxicity to human cells/organs/tissues; (ii) biocompatibility related to immune response; (iii) efficacy of drug or intervention against a target or disease.

There has been significant research into the development of biomimetic tissues, both as TE replacements and as *in vitro* models. The main challenges currently facing researchers is the introduction of complex and integral tissue systems which include vasculature, immune-competency and neural input. It is also apparent that for some systemic disease modelling, the TE of a single tissue or organ is not adequate. Therefore, approaches of TE multi-organ systems, including multi-organ-on-a-chip technology, have a role to play in understanding multi-organ communication with a focus on the importance of feedback. A focus going forward with this technology is how the introduction of organ-specific features can be incorporated, including differences in matrix stiffness, with a single fluid flow rate.

Key challenges include (i) the control of architectural features of tissues and organs. 3D printing and bio-printing go a significant way in solving this problem, however there remain limitations to controlling native proteins, their cross-linking and bonding between polymer chains. Another approach is the use of decellularized matrices, which can retain many architectural features. However the re-cellularisation process is challenging and in itself can result in significant remodelling of matrices and (ii) remodelling of the engineered tissue/organ by the host. An engineered tissue used to replace a damaged or diseased tissue may be functional, however following implantation into the host will be subject to remodelling, potential immune response and even wound healing processes as it integrates with surrounding tissues. Controlling these processes is difficult. Solutions may lie in the use of machine learning and artificial intelligence to predict problems, which will be patient specific to allow scientists and clinicians to troubleshoot.

Challenges with TE replacement body parts are significant, however the importance of realistic goals, with clearly defined limitations will help advancements in this field. There is also a clear cross-over here with the advent of more personalised medicine approaches with the use of patient-specific tissue models to predict therapeutic efficacy. Patient-derived xenografts are increasingly being used for personalised medicine approaches using animal models. Equally there is the consideration to use patient tissue-derived models or tissue models incorporating aspects of patient tissue to test specific therapies to predict best outcome.

The most challenging issues for TE for replacement body parts remains the reliable, reproducible and consistent manufacturing of TE products (https://www.fda.gov/vaccines-blood-biologics/tissue-tissue-products/fda-regulation-human-cells-tissues-and-cellular-and-tissue-based-products-hctps-product-list). Expertise on the optimal production strategies require multiple elements to sit together to ensure a deliverable and consistent manufacturing process which needs to be one that is rapidly scalable and ideally cost-efficient. A number of TE products generate insufficient revenue and thus limit their ability to be seen as viable opportunities for development by biotechnology and medical companies. There is an urgent need for innovative funding streams which incentivise manufacturers to develop such products and develop technologies to address challenges in TE.
